# SOCS1 Regulates Apoptosis and Inflammation by Inhibiting IL-4 Signaling in IL-1*β*-Stimulated Human Osteoarthritic Chondrocytes

**DOI:** 10.1155/2017/4601959

**Published:** 2017-03-08

**Authors:** Qiang He, Caihong Sun, Wei Lei, Jianbing Ma

**Affiliations:** ^1^Department of Orthopaedic Surgery, Honghui Hospital, Xi'an Jiaotong University, No. 555 East Youyi Road, Xi'an, Shaanxi 710054, China; ^2^Department of Informatics, 451 Hospital of PLA, No. 169 East Youyi Road, Xi'an, Shaanxi 710054, China; ^3^Institute of Orthopaedics, Xijing Hospital, Fourth Military Medical University, No. 169 West Changle Road, Xi'an, Shaanxi 710032, China

## Abstract

Recently, Suppressor of Cytokine Signaling 1 (SOCS1) was identified as a potential therapeutic target for osteoarthritis (OA) treatment. However, the mechanisms and signaling pathways of SOCS1 in the regulation of OA development are unclear. The purpose of the current study was to investigate whether interleukin- (IL-) 4 was involved in regulatory mechanism of SOCS1 in human osteoarthritic chondrocytes. First, IL-1*β* was used to stimulate human osteoarthritic chondrocytes isolated from the articular cartilage of OA patients undergoing total knee replacement. The protein and mRNA expression levels of SOCS1 were upregulated in IL-1*β*-stimulated human osteoarthritic chondrocytes compared with control cells. The knockdown of SOCS1 increased cell viability and inhibited cell apoptosis. It was also found that IL-4 expression was increased by SOCS1 silencing. Additionally, knockdown of IL-4 reduced cell viability and increased cell apoptosis of osteoarthritic chondrocytes transfected with SOCS1 siRNA. Moreover, the decreased expression of inflammatory factors induced by SOCS1 was enhanced by IL-4 knockdown. In conclusion, IL-4 signaling plays a crucial role in the regulatory functions of SOCS1 in apoptosis and inflammation in human osteoarthritic chondrocytes. These findings provide a potential therapeutic target for the clinical treatment of OA.

## 1. Introduction

Osteoarthritis (OA) is a common joint disease worldwide [1]. Nearly 250 million people in the world have OA of the knee and about half of individuals develop symptomatic knee OA by age 85 [2, 3]. Progressive destruction of articular cartilage (AC) is a hallmark of OA, and chondrocytes, the only resident cells in AC, play a key role via apoptosis, cytokine production, and matrix degeneration during the development of OA [4]. Thus, chondrocytes are regarded as target cells for OA therapy.

Suppressor of Cytokine Signaling 1 (SOCS1), a prototypical member of the SOCS family, contains a STAT-binding site within the promoter region and is located at 16p13 [5]. SOCS1 is widely expressed in various tissues and its expression is regulated by a variety of cytokines [6]. Initial studies on SOCS1 were largely focused on its negative regulation on cytokine signaling. Recently, more and more researchers have found that SOCS1 can also influence the transduction of proliferation signals and plays a role in the survival, differentiation, and transformation of cells. Emerging evidence has demonstrated that SOCS1 is an important regulator in human diseases, such as type II diabetes, cancer, and inflammatory diseases [6, 7]. Recently, Choi et al. [8] reported that the expression of SOCS1 is increased in OA cartilage and SOCS1 inhibits IL-1*β* signaling in chondrocytes. The inhibitory effects of SOCS1 were mediated by blocking p38, c-Jun N-terminal kinase (JNK), and nuclear factor kappaB activation and by downregulating transforming growth factor-*β*-activated kinase 1 expression. Alternatively, another study demonstrated that SOCS1 downregulates the p38-CREB-C/EBPbeta pathway resulting in increased expression of matrix metalloproteinases in chondrocytes [9]. Therefore, the exact mechanisms of SOCS1 in the development of OA remain to be clarified.

The interleukin- (IL-) 4/IL-4 receptor is a major system involved in the regulation of the function and integrity of cartilage chondrocytes in OA by inhibiting the release of inflammatory mediators [10]. IL-4 is an anti-inflammatory cytokine that suppresses the synthesis of proinflammatory cytokines, such as interleukin- (IL-) 1*β* and tumor necrosis factor- (TNF-) alpha, and inhibits many processes, such as the induction of apoptosis and inflammation [11–13]. A previous study has demonstrated that IL-4 expression is reduced in OA patients and suggested its role as a chondroprotective cytokine in OA [13]. SOCS1 is an important regulator of IL-4 signaling, and forced expression of SOCS-1 inhibits IL-4 signaling in macrophages [14, 15].

In the present study, we investigated the effects of SOCS1 on IL-1*β*-stimulated OA chondrocytes and explored whether IL-4 is involved in the protective effect of SOCS1. SOCS-1 expression was determined at the messenger RNA (mRNA) and protein levels in IL-1*β*-stimulated human articular chondrocytes. SOCS-1 expression was reduced by RNA interference to determine its effects on chondrocyte function. In addition, IL-1*β*-stimulated human osteoarthritic chondrocytes after SOCS1 knockdown were transfected with IL-4 siRNA to confirm whether IL-4 was involved in regulatory mechanism of SOCS1 in human osteoarthritic chondrocytes.

## 2. Materials and Methods

### 2.1. Isolation and Culture of Human Osteoarthritic Chondrocytes

All experiments were approved by the Honghui Hospital Human Research Ethics Committee (Permit Number: 2016–201). Primary human chondrocytes were isolated from the articular cartilage of OA patients (*n* = 12, mean age 65.8 ± 3.5 years, 10 female and 2 male, 12 knees with K/L grades of 3) undergoing total knee replacement, as previously described [16]. All patients provided informed written consent. Briefly, harvested cartilage was minced into small pieces and incubated in a 0.25% trypsin-containing solution for 2 h at 37°C. The pieces were then washed with phosphate-buffered saline (PBS) and incubated in 0.2% collagenase at 37°C overnight. After digestion, the chondrocytes were collected and cultured in Dulbecco's modified Eagle's medium (DMEM) (Gibco, Gaithersburg, USA) containing 10% fetal calf serum, 100 U/ml penicillin, and 100 *μ*g/ml streptomycin. Cells were plated in high density monolayers (2 × 10^6^ cells/well in 12 wells' culture plate) for 48 h. A careful attention was given to maintaining high density culture and to controlling chondrocytic phenotype.

### 2.2. Cell Treatment

For experiments with IL-1*β* stimulation, first-passage chondrocytes (2 × 10^6^ cells/well in 12 wells' culture plate) were replaced by serum-free DMEM for 24 h. Then the cells were treated with 10 ng/ml IL-1*β* [17] (Sigma-Aldrich, St. Louis, USA) for 24 h. The control group was untreated, but the medium was changed. Cells were harvested after stimulation.

### 2.3. RNA Interference

To inhibit SOCS1 expression, first-passage chondrocytes were cultured for three days to reach high cell density and transfected with SOCS1 siRNA or negative control using Lipofectamine 2000 (Invitrogen, Carlsbad, USA) in 24-well plates for 48 h. IL-4 siRNA was also transfected into cells using Lipofectamine 2000 (Invitrogen, Carlsbad, USA) for 48 h. The siRNA primer sequences were designed using Invitrogen Block-iT RNAi Designer (Table 1) and were as follows: SOCS1 siRNA_492: 5′-UCG CCC UUA GCG UGA AGA U dTdT-3′ (F), 5′-AUC UUC ACG CUA AGG GCG A dTdT-3′ (R); SOCS1 siRNA control_492: 5′-UCG AUU CUG CGA AGC CGA U dTdT-3′ (F), 5′-AUC GGC UUC GCA GAA UCG A dTdT-3′ (R); IL-4 siRNA_371: 5′-GCU GAU CCG AUU CCU GAA A dTdT-3′ (F), 5′-UUU CAG GAA UCG GAU CAG C dTdT-3′ (R).

### 2.4. Cell Viability

Cell viability was analyzed using cell counting kits (Sigma-Aldrich, USA). Cell viability was assayed by a 3-(4,5-dimethylthiazol-2-yl)-2,5-diphenyl tetrazolium bromide (MTT) assay as indicated time on normoxia or hypoxia after being treated with indicated experiments. In this assay, MTT was added to the cells at 37°C for 4 h. After incubation, MTT-containing medium was discarded and dimethyl sulfoxide (DMSO) was performed to dissolve formazan crystals. Optical densities (OD) were measured at 490 nm by Versamax microplate reader (Molecular Devices, Sunnyvale, CA, USA). Viability was normalized by OD value/cell number and 48 h normoxic culture treated with control RNA and si-control was denoted as 100%. Assays were repeated four times for each sample.

### 2.5. Cell Apoptosis

The apoptotic cells were measured by flow cytometry using an Annexin V-FITC/PI Apoptosis Detection Kit (Abcam, Cambridge, UK). The fluorescence intensity was detected at 488 nm by flow cytometry. Cells were harvested after indicated experiments and cell suspensions were fixed overnight with ice-cold 70% ethanol. Cells were sorted using a FACS Calibur Flow Cytometer (BD Medical Technology, USA) and analyzed using CellQuest software (BD Medical Technology, USA). The results were expressed as the percentage of apoptotic cells from the total cells.

### 2.6. Enzyme-Linked Immune-Sorbent Assay (ELISA)

The levels of IL-4 and TNF-alpha were measured using the Human IL-4 ELISA Kit or the Human TNF-alpha ELISA Kit, respectively, according to the manuals provided (Abcam, Cambridge, UK).

### 2.7. Western Blot

Total proteins were extracted from cells using the Tissue or Cell Total Protein Extraction Kit (Amresco, USA). The primary antibodies were purchased from Abcam (Abcam, Cambridge, UK). The proteins were separated by SDS-PAGE followed by electrotransfer to an NC membrane. The membrane were probed using antibodies against SOCS1 (1 : 1000), caspase-9 (1 : 1000), Bax (1 : 1000), Bcl-2 (1 : 1000), and IL-4 (1 : 500), followed by a horseradish peroxidase- (HRP-) conjugated secondary antibody (Abcam, Cambridge, UK). Bands were revealed using ECL reagent (Millipore, Boston, MA, USA) and recorded on X-ray films (Kodak, China). Densitometry analyses of each band were performed using the Gel Imaging System and Quantity One 4.62 software (Bio-Rad, Hercules, CA, USA).

### 2.8. Reverse Transcriptase-Polymerase Chain Reaction (RT-PCR)

Total RNA was extracted using TRIZOL Reagent (Invitrogen, Carlsbad, CA, USA) following the protocol recommended by the manufacturer. RNA was reverse-transcribed using SuperScript® First-Strand kit (Invitrogen). The first-strand cDNA was synthesized using 1 *μ*g of total RNA and SuperScript III Reverse Transcriptase (Invitrogen, Carlsbad, CA, USA). PCR amplification was performed using the PCR Amplification Kit (Takara Biotechnology, Dalian, China). The specific primers were designed using Primer Premier 6.0 software and synthesized by Sangon Biotech (Shanghai, China). The PCR products were checked on a 1% agarose gel and visualized using the Gel Imaging System of Bio-Rad Corp. (Bio-Rad, Hercules, CA, USA). Each band was analyzed using Quantity One 4.62 (Bio-Rad, Hercules, CA, USA).

### 2.9. Statistical Analysis

Data were reported as the mean ± SD of at least four replicates per group. Data were analyzed using SPSS13.0 (IBM, USA). Significant differences between means were assessed using ANOVA, followed by LSD multiple comparison tests. Differences were considered significant at *p* < 0.05.

## 3. Results

### 3.1. SOCS1 Was Upregulated in IL-1*β*-Stimulated Human Osteoarthritic Chondrocytes

**Figure 1:**
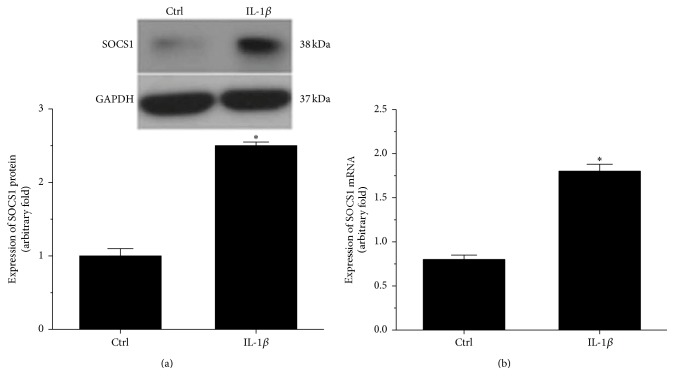
SOCS1 was upregulated in IL-1*β*-stimulated human osteoarthritic chondrocytes. SOCS1 protein and mRNA expression levels were detected by Western blotting (a) and RT-PCR (b), respectively. Ctrl: control (*n* = 4); IL-1*β*: human osteoarthritic chondrocytes were stimulated with 10 ng/ml IL-1*β* for 24 h (*n* = 4); ^*∗*^*p* < 0.05 versus Ctrl.

Western blotting and RT-PCR were used to evaluate the expression of SOCS1 in IL-1*β*-stimulated human osteoarthritic chondrocytes. As shown in Figure 1, the expression of SOCS1 was elevated in IL-1*β*-stimulated human osteoarthritic chondrocytes compared with control cells (*p* < 0.05, Figure 1(a)). A similar result was observed for the expression of* SOCS1* mRNA; stimulation with IL-1*β* induced an increase in the expression of* SOCS1* mRNA in human osteoarthritic chondrocytes compared with control cells (*p* < 0.01, Figure 1(b)).

### 3.2. Knockdown of SOCS1 Increased Cell Viability and Inhibited Apoptosis in IL-1*β*-Stimulated Human Osteoarthritic Chondrocytes

**Figure 2:**
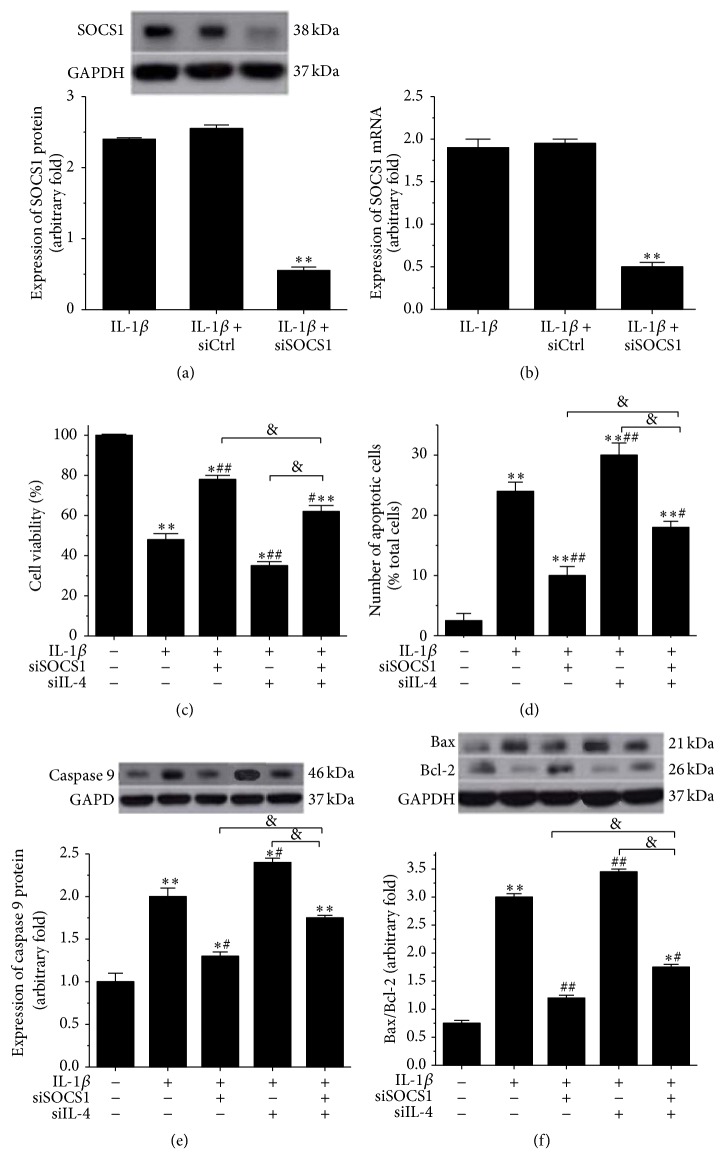
Knockdown of SOCS1 increased cell viability and inhibited apoptosis in IL-1*β*-stimulated human osteoarthritic chondrocytes transfected with and without siIL-4. The expression of SOCS1 at the protein and mRNA levels was detected by Western blotting (a) and RT-PCR (b), respectively, after transfection of SOCS1 siRNA or the negative control. IL-1*β*: human osteoarthritic chondrocytes were stimulated with 10 ng/ml IL-1*β* for 24 h (*n* = 4); IL-1*β* + siCtrl: human osteoarthritic chondrocytes were transfected with the negative control siRNA after stimulation with IL-1*β* (*n* = 4); IL-1*β* + siSOCS1: human osteoarthritic chondrocytes were transfected with SOCS1 siRNA after stimulation with IL-1*β* (*n* = 4). ^*∗∗*^*p* < 0.01 versus IL-1*β*. Cell viability was measured using cell counting kits (c). Cell apoptosis was detected by flow cytometry using an Annexin V-FITC/PI Apoptosis Detection Kit (d). The expression levels of caspase 9 (e) and Bax/Bcl-2 (f), which are hallmarks of cell apoptosis, were detected using Western blotting. IL-1*β*: 10 ng/ml IL-1*β* stimulation for 24 h (*n* = 4); siSOCS1: transfection with SOCS1 siRNA (*n* = 4); siIL-4: transfection with IL-4 siRNA (*n* = 4); “−”: no added, “+”: added. ^*∗*^*p* < 0.05 versus no addition, ^*∗∗*^*p* < 0.01 versus no addition, ^#^*p* < 0.05 versus only added IL-1*β*, ^##^*p* < 0.01 versus only added IL-1*β*, ^&^*p* < 0.05.

**Figure 3:**
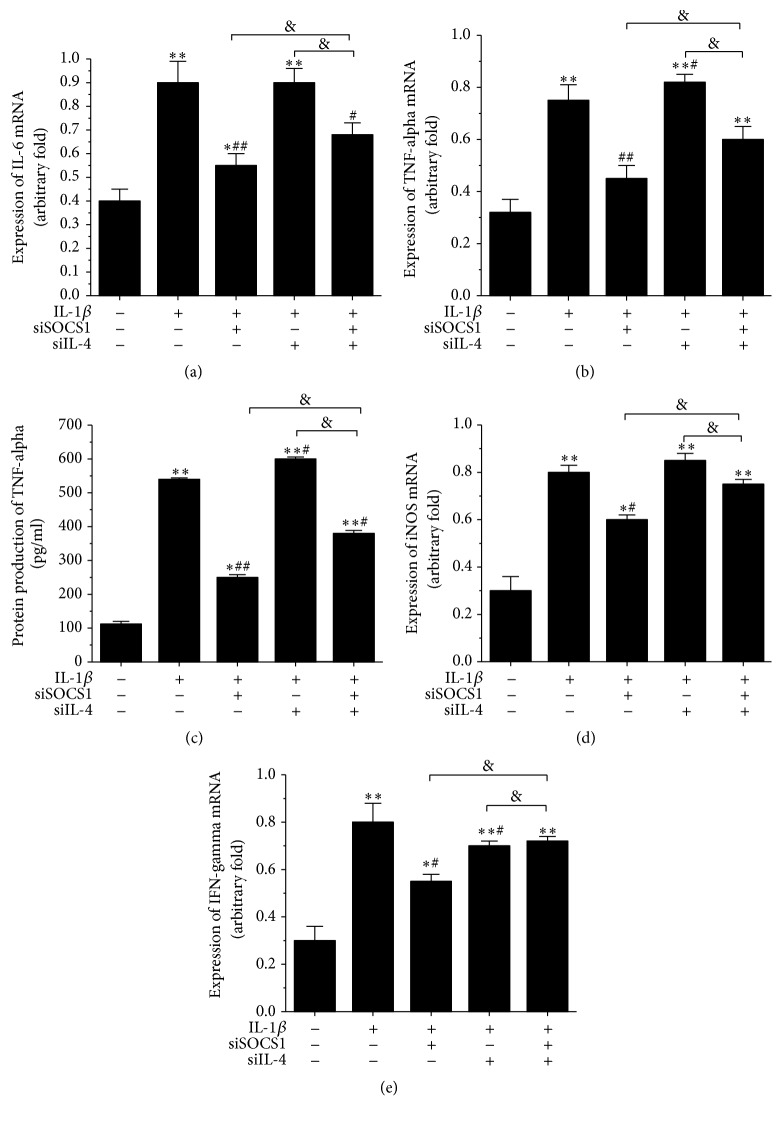
Knockdown of SOCS1 suppressed the production of inflammatory factors in IL-1*β*-stimulated human osteoarthritic chondrocytes transfected with and without siIL-4. Inflammatory factors, including IL-6 (a), TNF-alpha (b), iNOS (d), and IFN-gamma (e), were measured by RT-PCR. Protein production of TNF-alpha was detected in culture supernatants by ELISA (c). IL-1*β*: 10 ng/ml IL-1*β* stimulation for 24 h (*n* = 4); siSOCS1: transfection with SOCS1 siRNA (*n* = 4); siIL-4: transfection with IL-4 siRNA (*n* = 4); “−”: no added, “+”: added. ^*∗*^*p* < 0.05 versus no addition, ^*∗∗*^*p* < 0.01 versus no addition, ^#^*p* < 0.05 versus only added IL-1*β*, ^##^*p* < 0.01 versus only added IL-1*β*, ^&^*p* < 0.05.

To explore the effect of SOCS1 on the biological functions of osteoarthritic chondrocytes, we transfected SOCS1 siRNA or negative control siRNA into IL-1*β*-stimulated human osteoarthritic chondrocytes. The transfection results are displayed in Figures 2(a) and 2(b). The transfection of SOCS1 siRNA dramatically reduced the IL-1*β*-induced expression of* SOCS1* at the protein (*p* < 0.01, Figure 2(a)) and mRNA (*p* < 0.01, Figure 2(b)) levels. To estimate the effects of SOCS1 on cell viability and apoptosis in IL-1*β*-stimulated human osteoarthritic chondrocytes, we first estimated the percentage of viable cells among the total cells using cell counting kits. As shown in Figure 2(c), the IL-1*β*-induced reduction of cell viability was significantly elevated by the knockdown of SOCS1 (*p* < 0.05). Additionally, the apoptosis of human osteoarthritic chondrocytes was induced by IL-1*β* stimulation (*p* < 0.01), while the knockdown of SOCS1 notably suppressed IL-1*β*-induced cell apoptosis of human osteoarthritic chondrocytes (*p* < 0.01, Figure 2(d)). A similar result was obtained for the expression of caspase 9 and Bax/Bcl-2. As shown in Figure 2(e), IL-1*β*-induced expression of caspase 9 was reduced by the knockdown of SOCS1 (*p* < 0.05, Figure 2(e)). Moreover, the ratio of Bax to Bcl-2 was increased by IL-1*β* stimulation (*p* < 0.01), while the knockdown of SOCS1 inhibited the IL-1*β*-induced increase in the ratio of Bax to Bcl-2 (*p* < 0.01, Figure 2(f)). Besides, knockdown of SOCS1 increased cell viability, inhibited apoptosis (Figure 2), and suppressed the production of inflammatory factors (Figure 3) in IL-1*β*-stimulated human osteoarthritic chondrocytes which were transfected with siIL-4.

We transfected with IL-4 siRNA to assay cell viability, apoptosis, and the expression of inflammatory factors to investigate whether IL-4 was involved in IL-1*β*-stimulated human osteoarthritic chondrocytes. The knockdown of IL-4 decreased cell viability (*p* < 0.01, Figure 2(c)) and increased the number of apoptotic cells (*p* < 0.01, Figure 2(d)) in IL-1*β*-stimulated chondrocytes. In addition, the knockdown of IL-4 increased the protein expression of caspase 9 (*p* < 0.05, Figure 2(e)) and Bax/Bcl-2 ratio (*p* < 0.01, Figure 2(f)). The expression of inflammatory factors, including TNF-alpha (*p* < 0.05, Figures 3(b) and 3(c)) and IFN-gamma (*p* < 0.05, Figure 3(e)), increased after IL-1*β*-stimulated chondrocytes transfected with IL-4 siRNA.

To confirm whether IL-4 was involved in the regulatory mechanism of SOCS1 on human osteoarthritic chondrocytes, IL-1*β*-stimulated human osteoarthritic chondrocytes after SOCS1 knockdown were transfected with IL-4 siRNA to assay cell viability, apoptosis, and the expression of inflammatory factors. As shown in Figure 2, the knockdown of IL-4 dramatically reduced the SOCS1 siRNA-induced enhancement of cell viability (*p* < 0.05, Figure 2(c)) and increased the SOCS1 siRNA-induced reduction of apoptosis (*p* < 0.05, Figure 2(d)) in IL-1*β*-stimulated human osteoarthritic chondrocytes. Moreover, the knockdown of IL-4 markedly enhanced the SOCS1 siRNA-induced reduction of the expression of caspase 9 (*p* < 0.05, Figure 2(e)) and increased the ratio of Bax to Bcl-2 (*p* < 0.05, Figure 2(f)) in IL-1*β*-stimulated human osteoarthritic chondrocytes. Additionally, the knockdown of IL-4 significantly increased the SOCS1 siRNA-induced reduction in the expression of inflammatory factors, including IL-6 (*p* < 0.05, Figure 3(a)), TNF-alpha (*p* < 0.05, Figures 3(b) and 3(c)), iNOS (*p* < 0.05, Figure 3(d)), and IFN-gamma (*p* < 0.01, Figure 3(e)) in IL-1*β*-stimulated human osteoarthritic chondrocytes. Therefore, these data indicated that IL-4 was involved in the regulatory mechanism of SOCS1 on cell viability, apoptosis, and inflammatory responses of human osteoarthritic chondrocytes.

### 3.3. Knockdown of SOCS1 Suppressed the Expression of Inflammatory Cytokines in IL-1*β*-Stimulated Human Osteoarthritic Chondrocytes

It has been demonstrated that inflammatory factors, including IL-6 and TNF-alpha, are involved in the pathophysiology of osteoarthritis [18]. Moreover, proinflammatory cytokines (IL-6, TNF-alpha, and IFN-gamma) influence chondrocyte viability (necrosis/apoptosis), proliferation, and nitric oxide (NO) production [12]. Therefore, we investigated the effect of SOCS1 on the expression of inflammatory cytokines in IL-1*β*-stimulated human osteoarthritic chondrocytes. As shown in Figure 3, IL-1*β* stimulation increased the expression of inflammatory cytokines, including IL-6 (*p* < 0.01, Figure 3(a)), TNF-alpha (*p* < 0.01, Figures 3(b) and 3(c)), iNOS (*p* < 0.01, Figure 3(d)), and IFN-gamma (*p* < 0.01, Figure 3(e)), compared with the controls. Knockdown of SOCS1 reduced the IL-1*β*-induced elevation of the production of inflammatory factors. These data indicated that SOCS1 was involved in the regulation of inflammatory responses of human osteoarthritic chondrocytes and may play a proinflammatory role in chondrocytes.

### 3.4. IL-4 Was Downregulated in IL-1*β*-Stimulated Human Osteoarthritic Chondrocytes and Upregulated by SOCS1 Silencing

**Figure 4:**
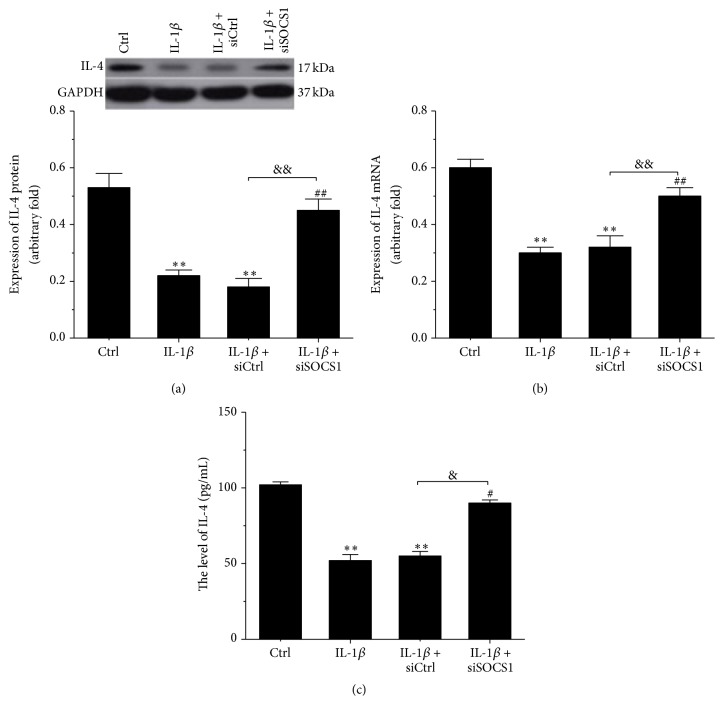
Knockdown of SOCS1 upregulated IL-1*β*-induced IL-4 expression in human osteoarthritic chondrocytes. Expression of IL-4 at the protein (a) and mRNA (b) levels was detected using Western blotting and RT-PCR, respectively. Additionally, the concentration of IL-4 was measured by ELISA (c). Ctrl: control (*n* = 4); IL-1*β*: human osteoarthritic chondrocytes were stimulated with 10 ng/mL IL-1*β* for 24 h (*n* = 4); IL-1*β* + siCtrl: human osteoarthritic chondrocytes were transfected with the negative control after IL-1*β* stimulation (*n* = 4); IL-1*β* + siSOCS1: human osteoarthritic chondrocytes were transfected with SOCS1 siRNA after IL-1*β* stimulation (*n* = 4). ^*∗∗*^*p* < 0.01 versus Ctrl, ^#^*p* < 0.05 versus IL-1*β*, ^##^*p* < 0.01 versus IL-1*β*, ^&^*p* < 0.05, ^&&^*p* < 0.01.

Few studies have shown that IL-4 is associated with the pathological process of osteoarthritis [13, 19]; accordingly, we mainly explored the expression of IL-4 and the relationship between IL-4 and SOCS1 in IL-1*β*-stimulated human osteoarthritic chondrocytes. As shown in Figure 4, the expression of the IL-4 protein was observably reduced in IL-1*β*-stimulated human osteoarthritic chondrocytes compared with controls (*p* < 0.01, Figure 4(a)). IL-1*β* induced a reduction in the expression of IL-4 mRNA, which was elevated by the knockdown of SOCS1 (*p* < 0.01, Figure 4(b)). Knockdown of SOCS1 dramatically increased the IL-1*β*-induced reduction in the concentration of IL-4 (*p* < 0.05, Figure 4(c)). Therefore, we concluded that IL-4 participated in the effect of SOCS1 on the biological function of osteoarthritic chondrocytes.

## 4. Discussion

OA is a degenerative joint disease characterized by the loss of chondrocyte function caused by various risk factors, including genetic and environmental factors and aging [20, 21]. The loss of chondrocyte function and proinflammatory cytokines, such as IL-6, IL-8, IL-1*β*, and TNF-alpha, play crucial roles in the initiation and progression of OA, and thus the inhibition of these factors is an important issue for the treatment of OA [22, 23]. In this study, we found that SOCS1 silencing regulates apoptosis and inflammatory cytokine production in human osteoarthritic chondrocytes, probably by activating IL-4 signaling.

Studies have reported inconsistent results on the expression of the SOCS1 in OA cartilage or chondrocytes. de Andrés et al. [24] revealed that the SOCS1 and SOCS3 mRNA levels were similar in hip OA and normal chondrocytes. Van De Loo et al. [25] reported that the expression of SOCS1 mRNA was not enhanced in chondrocytes obtained from OA cartilage, compared with normal chondrocytes. In contrast, Choi et al. [8] showed that the expression of SOCS1 mRNA in OA cartilage was significantly higher than that of healthy cartilage and indicated that the upregulated expression of SOCS1 was mostly observed in areas of severely damaged cartilage. In our study, chondrocytes were also isolated from severely damaged cartilage (K/L grades of 3). In addition, the mRNA expression of SOCS1 was inducible by IL-1*β*, consistent with the observation by Van De Loo et al. [25] and Choi et al. [8]. They indicated that IL-1*β* stimulated SOCS1 mRNA expression in a dose-dependent pattern. Actually, IL-1*β* played important role in OA phenotype induction and maintenance. A recent study showed that IL-1*β* induced ultrastructural and cytoskeletal modifications in normal and OA chondrocytes [26]. Therefore, the differences between the findings may be primarily related to differences in the severity of OA and OA phenotype induction by IL-1*β*. Indeed, previous studies demonstrated that expression of functional genes such as collagen type II and GAG decreased with severe degeneration of OA cartilage [27].

The proinflammatory cytokine IL-1*β* is elevated in OA patients and plays a critical role in the pathogenesis of OA [28]. IL-1*β* is widely used to stimulate the expression of inflammatory cytokines implicated in OA pathology and as a chondrocyte apoptosis-inducing agent [29–31]. It has been reported that IL-1*β*-inducible SOCS1 acts as a negative regulator of the IL-1*β*-induced synthesis of matrix-degrading enzymes such as MMP-1, MMP-3, MMP-13, and ADAMTS-4 in OA cartilage [8]. In the present study, we explored the effect of SOCS1 on IL-1*β*-induced inflammatory cytokine release and apoptosis and found that SOCS1 regulates cell apoptosis and the expression of inflammatory cytokines. These findings indicate that the knockdown of SOCS1 could inhibit chondrocyte apoptosis and the release of inflammatory cytokines induced by IL-1*β*. Taken together with the previous results, it is believed that SOCS1 plays a novel role in the pathogenesis of OA. Although the roles of the MAPK and NF-*κ*B pathways have been established in the regulatory activity of SOCS1 in the development of OA, the multiple and complex mechanisms are far from being understood.

The chondroprotective cytokine IL-4 is expressed at lower levels in OA cartilage than normal cartilage and is believed to be a pivotal anabolic cytokine in cartilage, with the ability to regulate OA chondrocyte production of chemokines stimulated by IL-1*β* [13]. The results of the present study also showed a decrease in IL-4 expression in IL-1*β*-stimulated chondrocytes, which confirmed the crucial role of IL-4 in the development and pathophysiology of OA. Losman et al. [14] reported that SOCS-1 is a potent inhibitor of IL-4 signaling in M2 cells, while Woodward et al. indicated that IL-4 is not mediated by SOCS1 in monocytes and macrophages [32]. Thus, the relationship between SOCS1 and IL-4 is complex and depends on cell type. Multiple mechanisms have been implicated in the regulation of SOCS1 via the IL-4 signaling pathway. Venkataraman et al. has suggested that SOCS-1 may be important in the ability of IFN-gamma to suppress IL-4 biological functions [33]. Moreover, IL-6 inhibits IFN-gamma receptor-mediated signaling [34], suggesting that IL-6 is associated with the regulatory mechanism of SOCS1 on IFN-gamma signaling pathway-induced inhibition of IL-4 biological functions. Other work [33] has demonstrated that SOCS-1 inhibits JAK1/STAT6 during IL-4 signaling by preventing the phosphorylation of JAK1/STAT6 and blocking IL-4-induced gene expression. However, whether these mechanisms are also applicable to the chondroprotective function of IL-4 signaling in OA cartilage requires further investigation. Our study clarified the connection between increased SOCS1 and decreased IL-4 in IL-1*β*-stimulated chondrocytes for the first time. These results implied that the silencing of SOCS1 significantly upregulated IL-4 expression, which suggests a negative effect of SOCS1 on IL-4 signaling. Next, we examined whether SOCS1 regulates IL-1*β*-induced chondrocyte apoptosis and inflammatory cytokine production via IL-4 signaling, at least in part. A role of IL-4 in the regulatory mechanism of SOCS1 was observed in IL-1*β*-stimulated human osteoarthritic chondrocytes.

## 5. Conclusions

In conclusion, this study demonstrated that IL-1*β* stimulation increased the expression of SOCS1, and the knockdown of SOCS1 increased cell viability and inhibited cell apoptosis and expression of inflammatory cytokines. Furthermore, we also found that SOCS1 silencing regulated apoptosis and inflammatory cytokine production, probably by activating IL-4 signaling. To the best of our knowledge, this study provides the first evidence that SOCS1 is a potential therapeutic target for the treatment of OA. However, further in vivo experiments are needed to verify this inference. In the future, we will investigate the detailed regulatory mechanisms and relevant signaling molecules that mediate the effects of SOCS1 on IL-4 signaling in OA chondrocytes in vivo and in vitro.

## Figures and Tables

**Table 1:** Primer sequences.

	Forward (5′-3′)	Reverse (5′-3′)	Length of production (bp)	Efficiency (%)	Tm (°C)	Accession number
SOCS1	CTTCCTCCTCTTCCTCCTC	GCCATCTTCACGCTAAGG	286	93.7	50.8	NM_003745.1
IL-6	GTGAGGAACAAGCCAGAG	TGACCAGAAGAAGGAATGC	283	101.2	50.1	NM_000600.4
TNF-alpha	TCCAGACTTCCTTGAGACA	GGCGATTACAGACACAACT	369	104.8	50.7	NM_000594.3
iNOS	CTCAGCAAGCAGCAGAAT	CGTCAGTTGGTAGGTTCC	402	103.5	49.8	L24553.1
IFN-gamma	GGTTCTCTTGGCTGTTACT	ATGTCTTCCTTGATGGTCTC	251	98.5	50.1	NM_000619.2
IL-4	CCTCTGTTCTTCCTGCTAG	CTCTGGTTGGCTTCCTTC	365	99.3	51.6	NM_000589.3
GAPDH	GGCTCTCCAGAACATCATC	TCTTCCTCTTGTGCTCTTG	448	103.9	50.5	NM_001256799.2
